# Cancer risks in a population-based study of agricultural workers: results from the Taiwan’s Farmers and Health Cohort study

**DOI:** 10.5271/sjweh.4106

**Published:** 2023-09-01

**Authors:** Wei-Liang Chen, Gwan-Ling Lin, Yu-Jen Lin, Ting-Yao Su, Chung-Ching Wang, Wei-Te Wu

**Affiliations:** 1 Division of Family Medicine, Department of Family and Community Medicine, Tri-Service General Hospital, National Defense Medical Center, Taipei, Taiwan.; 2 Division of Occupational Medicine, Department of Family Medicine and Community Medicine, Tri-Service General Hospital, National Defense Medical Center, Taipei, Taiwan.; 3 Department of Nursing, Cardinal Tien Junior College of Healthcare and Management, New Taipei City, Taiwan.; 4 National Institute of Environmental Health Sciences, National Health Research Institutes, Miaoli, Taiwan.; 5 Institute of Environmental and Occupational Health Sciences, National Yang-Ming Chiao Tung University, Taipei, Taiwan.

**Keywords:** exposure, national-based database, Taiwan

## Abstract

**Objective:**

The purpose of this study was to assess cancer risk among agricultural workers compared to the general population.

**Methods:**

The study utilized data from Farmers’ Health Insurance (FHI) in Taiwan, which enrolled agricultural workers (N=1 175 149). The enrolled workers were matched to a general population (N=1 175 149) of the same age, gender, township, and enrollment year. The study population was linked to the National Cancer Registry to identify new cancer cases between 2000 and 2018. The Cox proportional hazards model was used to estimate the hazard ratio and 95% confidence interval for outcomes.

**Results:**

During the study period, 136 913 new cancers among agricultural workers were identified. The study found that male farmers had an increased cancer risk, including lymphocytic leukemia, chronic myelogenous leukemia, non-Hodgkin’s lymphoma (NHL), oral cancer, lip cancer, esophagus cancer, rectum and rectosigmoid junction cancer, liver and intrahepatic bile duct cancer, lung cancer, trachea and bronchi cancer, and other non-melanoma skin cancer, even when considering the latency period. Female farmers had an elevated risk of multiple myeloma and other non-melanoma skin cancer. Moreover, only lymphoma, NHL, other lymphoid, and multiple myeloma, were both found to occur at different insurance periods.

**Conclusions:**

This study provides farmer cancer patterns and risk, adding to the evidence that farmers are at increased risk of certain types of cancer, especially for hematological cancers. As exposure varies by farm operation type, individual farmer exposure may vary widely. Further understanding of the complex relationship between occupational exposure, environmental factors, and lifestyle factors is needed.

It has been estimated that 880 million persons were employed in the agriculture sector globally in 2019 (1). Agricultural workers represent a unique population whose operations are exposed to a variety of occupational hazards, such as outdoor exposure to ultraviolet radiation (UVR) and high temperature (2, 3), as well as exposure to microbial agents, endotoxins (4, 5), pesticides for disease and insect control, herbicides exposure for weed control (6, 7), and diesel exhaust fumes, solvents, metals, grain dust and crystalline silica (5, 8). Among them, UVR, crystalline silica and diesel exhaust were identified as the International Agency for Research on Cancer (IARC) group 1 human carcinogens (9).

Agriculture is one of the largest sectors in Taiwan, employing over 1 million workers. Due to Taiwan’s hot and humid climate, pests and diseases are prevalent, and chemical pesticides have been extensively used to maintain food production and profitability. Taiwan’s agricultural characteristics are similar to those of other Asian countries, including small-scale intensive farming, an aging workforce, family labor, and farmland in proximity to residential areas. While there have been some surveys on special occupational disease among Taiwanese farmers, such as onion fungal corneal ulcers (10–12), there is a lack of long-term studies to understand the cancer risk profile of this population.

The relationship between agriculture and cancer has received considerable attention and has given rise to a series of meta-analyses (13–20). In general, the overall risk of cancer in agricultural populations in Western countries is lower than in the general population, due to lifestyle factors such as lower smoking rates, higher levels of occupational physical activity, and exposure to livestock in relation with endotoxin (13–17). In addition, subcategories of cancer such as lymphoma and hematopoietic cancers, melanoma, lip cancer, prostate cancer and brain cancer are at excess risk (13, 14, 18–20). Despite an increasing body of evidence suggesting a distinct cancer incidence pattern among agricultural workers and farmers, meta-analyses have produced inconsistent and highly heterogeneous results (13, 14, 18, 19). The reasons for variability arising may be from the type of farming in each region, the study period, and the use of mortality or morbidity as the primary outcome. Moreover, cancer incidence rates and patterns vary widely between racial/ethnic groups and countries, but past studies have focused on Western and male populations, and less on Asian groups (16, 21, 22). To date, IARC has coordinated the formation of a consortium of agricultural cohorts (AGRICOH). The consortium includes 26 prospective cohorts from 12 countries, but there is still only one Korean cohort representing Asian countries (23).

Therefore, this study utilizes both the national cancer registration system and a farmer registration system to track the occurrence of cancer among farmers over time and mitigate the issue of long latency between exposure and cancer development in the agricultural environment. The objective is to assess the cancer risk of agricultural workers in Taiwan compared to the general population.

## Methods

### Study population

The study’s subjects were agricultural workers who participated in Taiwan’s state-run Farmers’ Health Insurance (FHI) program since 1985. The FHI is a government-sponsored health and social insurance program for farmers. To ensure that the FHI primarily serves full-time farmers, participants must meet strict criteria, including being >15 years of age, not engaging in full-time employment off-farm, working on a farm ≥90 days per year, and having ≥0.1 hectares of farmland (24). Between 2000 and 2009, the whole agricultural workers cohort comprised 1 232 604 farmers, including 718 881 men and 513 723 women, with an average age of 50.3 [standard deviation (SD)12.4] years of enrollment. The National Health Insurance Research Database (NHIRD) covered 99.9% of the Taiwanese population by the end of 2014, which comprises approximately 23 million people (25). For the control group, we selected a representative sample from the NHIRD, matched to the study cohort for the same age, gender, and township residence. The study population was linked to the National Cancer Registry (TCR) to identify new cancer cases from 2000 to 2018. We excluded cases where the cancer occurred prior to the date of enrollment (N=57 455). Finally, the study was conducted on a total of 2 350 298 individuals, including agricultural workers (N=1 175 149) and the general population (N=1 175 149), with an average age of 47.0 years (SD 16.0) of enrollment. The age discrepancy (47.0 versus 50.3 years old) was attributed to the exclusion of cases where cancer occurred prior to the date of enrollment. Approximately 4.6% of the total population (57 455 individuals) were excluded due to this criterion, and they tended to have relatively higher ages.

### Data sources for outcomes

The study population was linked to the TCR to track the occurrence of initial cancer diagnoses (non-metastatic cases). The TCR captures 98.1% of cancer cases in Taiwan in 2020 and maintains high data quality, with a death certificate only of 0.70% and cases microscopically verified of 93.72% (26, 27). Additionally, we utilized the Taiwan Death Register to track the death records. Mortality data was obtained from accurate and complete mortality registries in Taiwan, where all deaths are mandatorily registered and death certificates are completed by physicians (28). These sources provided us with essential details such as dates of death and underlying cause-of-death for each deceased individual.

Researchers used individual’s national ID number to identify newly cancer cases and information about cancer among the cohort and control population from the TCR from 1 January 2000 to 31 December 2018. The target cancers were based on the International Classification of Disease for Oncology 3rd edition (ICD-O-3) and the 2008 WHO classification of lymphoid neoplasms and beyond. Cases with a behavior code of 2 (in situ) in the ICD-O-3 were included in this study. Meanwhile, the histology (morphology) of the malignancies was identified according to the ICD-O-3 (supplementary material, www.sjweh.fi/article/4106, table S1).

### Statistical analysis

Participants who survived and did not have cancers before the cut-off date (31 December 2018) contributed to the person-year (PY) time between the initial date of insurance and cut-off date. Participants who survived and had cancers contributed to the PY time between the initial date of insurance and the cancer diagnosis date. Those who died and did not have cancers before the cut-off date contributed to the PY time between their initial date of insurance and date of death. The Cox proportional hazards model was used to assess the hazard ratio (HR) and 95% confidence interval (CI) for cancer, and stratification analysis was analysis conducted by gender and insurance period. The latency period of solid tumors is usually 10–12 years and hematological tumors is usually two years (29). Therefore, we excluded participants who had been newly diagnosed with solid tumors and had not been insured for more than ten years, as well as participants who had been newly diagnosed with hematological tumors and had not been insured for at least two years for sensitivity analysis. The study adjusted for several area-level potential predictors of cancer, including smoking rate, drinking rate, betel nut chewing rate, number of patients served by physicians, number of chronic beds per 10 000 population, mammography screening rate (only in breast cancer analysis), oral mucosa examination utilization rate (only in oral and oropharyngeal cancer analysis), and ultraviolet ray exceedance rate (only in non-melanoma cancer analysis) for 21 counties and cities (supplementarey table S2). The data was obtained from the Health Promotion Administration, Ministry of Health and Welfare (Taiwan), except for the ultra-violet index data, which was obtained from the Central Weather Bureau. The ultraviolet ray exceedance rate was calculated as the cumulative number of days with ultra-violet index≥8 for each county and city each year. The analysis was performed using SAS software (version 9.4; SAS Institute, Cary, NC, USA).

## Results

Cohort characteristics of the 1 175 149 farmers and 1 175 149 general population controls matched with age, gender, and area of insurance are presented in table 1. There were 136 913 cases of cancer among farmers and 130 248 cases of cancer among the general population controls. Their mean age was 47.0 (SD 16.0) years of enrollment, of which 687 138 (58.5%) were males and 488 011 (41.5%) were females.

**Table 1:** Distribution of agricultural workers and general population controls by age, gender, and area of insurance. [SD=standard deviation.]

		Agricultural workers (N=1 175 149) ^a, b^			General population controls (N=1 175 149) ^a^	
		N	%		N	%
Cancer cases		136 913	11.7		130 248	11.1
Sex						
	Male	687 138	58.5		687 138	58.5
	Female	488 011	41.5		488 011	41.5
Age (year)						
	20–29	173 782	14.8		173 782	14.8
	30–39	259 325	22.1		259 325	22.1
	40–49	245 807	20.9		245 807	20.9
	50–59	209 422	17.8		209 422	17.8
	60–69	176 582	15.0		176 582	15.0
	≥70	110 231	9.4		110 231	9.4
Insurance Area						
	Changhua County	138 443	11.8		138 443	11.8
	Tainan city	127 075	10.8		127 075	10.8
	Taichung city	125 266	10.7		125 266	10.7
	Pingtung County	104 715	8.9		104 715	8.9
	Kaohsiung city	100 489	8.6		100 489	8.6
	Yunlin County	87 446	7.4		87 446	7.4
	Chiayi County	81 691	7.0		81 691	7.0
	Nantou County	76 228	6.5		76 228	6.5
	Taoyuan City	66 049	5.6		66 049	5.6
	Miaoli County	54 142	4.6		54 142	4.6
	New Taipei City	51 633	4.4		51 633	4.4
	Yilan County	34 751	3.0		34 751	3.0
	Hsinchu County	30 394	2.6		30 394	2.6
	Taitung County	29 391	2.5		29 391	2.5
	Hualien County	26 732	2.3		26 732	2.3
	Chiayi City	12 282	1.0		12 282	1.0
	Taipei City	11 523	1.0		11 523	1.0
	Penghu County	7657	0.7		7657	0.7
	Hsinchu city	4920	0.4		4920	0.4
	Kinmen County	3025	0.3		3025	0.3
	Keelung city	1176	0.1		1176	0.1
	Lianjiang County	121	<0.1		121	<0.1

^a^ Mean age at enrollment47 (SD 16) years. ^b^ Insurance seniority 16.1 (SD 7.2) years.

Supplementary table S3 presents the numbers of cancer cases and corresponding HR and 95% CI for various cancer types among Taiwanese farmers. The study found that, compared to the general population, farmers had significantly increased risks of several hematological cancers, including lymphocytic leukemia, acute lymphocytic leukemia, other leukemia, lymphoma, non-Hodgkin’s lymphoma (NHL), and multiple myeloma.

In terms of solid tumors, this study found that farmers had significantly increased risks of certain types of cancer, including oral, lip, esophagus, rectum and rectosigmoid junction, liver and intrahepatic bile duct, lung, trachea and bronchi, melanoma of skin, and other non-melanoma skin cancer. After considering the 10-year latency period in solid tumors and 2-year latency period in hematological tumors, the aforementioned cancer types showed significantly elevated risks, except for acute lymphocytic leukemia, trachea and bronchi, and melanoma of skin (supplementary table S3).

When the latency period is considered, male farmers had an elevated risk of developing certain types of cancer, including overall cancer (Model 2: HR 1.11, 95% CI 1.10–1.13), lymphocytic leukemia (Model 2: HR 1.12, 95% CI 1.04–1.21), chronic myelogenous leukemia (CML) (Model 2: HR 1.26, 95% CI 1.03–1.55), NHL (Model 2: HR 1.10, 95% CI 1.01–1.20), oral cancer, lip cancer, esophagus cancer, rectum and rectosigmoid junction cancer, liver and intrahepatic bile duct cancer, lung cancer (Model 2: HR 1.17, 95% CI 1.13–1.22), trachea and bronchi cancer (Model 2: HR 1.36, 95% CI 1.00–1.86), and other non-melanoma skin cancer (Model 2: HR 1.22, 95% CI 1.14–1.31). Female farmers had an elevated risk of multiple myeloma (Model 2: HR 1.29, 95% CI 1.02–1.63), and other non-melanoma skin cancer (Model 2: HR 1.20, 95% CI 1.09–1.32) (table 2).

**Table 2:** Hazard ratio (HR) and 95% confidence intervals (CI) for cancer types between agricultural workers by gender.

Sites/Types		Male							Female					
		Model 1 ^a^				Model 2 ^b^			Model 1 ^a^				Model 2 ^b, c^	
		Cases	HR	95% CI		HR	95% CI		Cases	HR	95% CI		HR	95% CI
Overall Cancer		90 678	1.10	1.09‒1.11		1.11	1.10‒1.13		46 235	0.97	0.96‒0.98		0.97	0.95‒0.99
Malignant neoplasm of lymphatic and haemopoietic tissue														
	Lymphocytic leukemia	1652	1.11	1.04‒1.19		1.12	1.04‒1.21		732	1.06	0.95‒1.17		1.07	0.96‒1.19
	Acute myelogenous leukemia (AML)	633	1.10	0.99‒1.23		1.07	0.95‒1.20		305	1.14	0.96‒1.34		1.14	0.96‒1.35
	Acute lymphoblastic leukemia (ALL)	45	1.36	0.87‒2.13		1.31	0.83‒2.07		14	0.50	0.26‒0.95		0.50	0.26‒0.95
	Chronic myelogenous leukemia (CML)	223	1.24	1.02‒1.51		1.26	1.03‒1.55		79	0.94	0.69‒1.27		0.97	0.71‒1.33
	Chronic myelogenous leukemia (CLL)	169	0.97	0.78‒1.20		1.07	0.85‒1.34		63	0.92	0.65‒1.30		0.90	0.64‒1.29
	Other leukemia	582	1.11	0.98‒1.25		1.13	0.99‒1.28		271	1.12	0.94‒1.33		1.13	0.95‒1.35
	Lymphoma	2578	1.06	1.00‒1.12		1.08	0.99‒1.17		1340	1.09	1.00‒1.17		1.11	0.99‒1.24
	Hodgkin’s lymphoma	76	0.78	0.58‒1.05		0.62	0.39‒0.98		42	1.35	0.85‒2.14		2.11	0.95‒4.66
	Non-Hodgkin’s lymphoma (NHL)	2502	1.07	1.01‒1.13		1.10	1.01‒1.20		1298	1.08	1.00‒1.17		1.09	0.98‒1.22
	B-cell lymphomas	1503	1.04	0.97‒1.12		1.09	0.99‒1.21		841	1.09	0.99‒1.21		1.07	0.93‒1.22
	Natural killer (NK)/T-cell lymphoma	275	1.06	0.89‒1.25		0.99	0.76‒1.28		102	0.92	0.71‒1.21		0.94	0.63‒1.40
	Other lymphoid	724	1.15	1.03‒1.28		1.18	0.99‒1.39		355	1.10	0.94‒1.27		1.23	0.99‒1.53
	Multiple myeloma	555	1.17	1.04‒1.33		1.16	0.97‒1.39		283	1.19	1.00‒1.41		1.29	1.02‒1.63
Malignant neoplasm of lip, oral cavity and pharynx														
	Oral	7688	1.23	1.19‒1.27		1.29	1.23‒1.35		506	0.92	0.82‒1.04		0.93	0.78‒1.10
	Lip	432	1.38	1.20‒1.60		1.55	1.27‒1.89		49	1.11	0.74‒1.66		1.13	0.64‒1.98
	Larynx	1066	1.01	0.93‒1.10		1.11	0.98‒1.26		24	0.61	0.37‒1.02		0.59	0.27‒1.28
	Major salivary glands	215	1.02	0.84‒1.23		0.94	0.71‒1.25		99	0.89	0.68‒1.17		0.78	0.53‒1.15
Malignant neoplasm of digestive organs and peritoneum														
	Esophagus	3645	1.18	1.13‒1.24		1.29	1.21‒1.38		145	0.79	0.63‒0.98		0.79	0.58‒1.07
	Stomach	4462	1.03	0.99‒1.07		1.08	1.01‒1.15		1653	1.03	0.96‒1.11		0.97	0.88‒1.08
	Colon (excluding rectum)	7607	0.96	0.93‒0.99		0.95	0.91‒0.99		3942	0.96	0.92‒1.01		0.98	0.92‒1.04
	Rectum and rectosigmoid junction	5840	1.14	1.10‒1.18		1.12	1.06‒1.18		2357	1.04	0.98‒1.10		1.03	0.95‒1.12
	Sigmoid colon	3498	1.01	0.96‒1.06		0.98	0.92‒1.05		1486	0.94	0.88‒1.01		0.96	0.87‒1.05
	Anus, anal canal, and anorectum	52	0.53	0.38‒0.74		0.70	0.42‒1.16		40	0.74	0.49‒1.11		0.75	0.43‒1.32
	Liver and intrahepatic bile duct	15 480	1.17	1.15‒1.20		1.19	1.15‒1.23		4186	0.97	0.93‒1.01		1.00	0.95‒1.07
Malignant neoplasm of respiratory and intrathoracic organs														
	Lung	13 210	1.15	1.12‒1.18		1.17	1.13‒1.22		4582	0.96	0.92‒1.00		0.98	0.93‒1.04
	Trachea and bronchi	306	1.26	1.06‒1.49		1.36	1.00‒1.86		42	0.77	0.52‒1.16		0.63	0.31‒1.30
Malignant neoplasm of bone, connective tissue, skin and breast														
	Bones, joints, and articular cartilage	78	1.12	0.81‒1.54		1.34	0.81‒2.21		43	1.07	0.70‒1.65		1.25	0.65‒2.41
	Connective, subcutaneous and other soft tissues	373	0.91	0.79‒1.05		0.87	0.70‒1.07		218	1.12	0.93‒1.36		1.21	0.91‒1.60
	Melanoma of skin	137	1.20	0.94‒1.54		1.14	0.79‒1.66		86	1.82	1.27‒2.60		1.71	1.00‒2.93
	Other non-melanoma skin	3269	1.20	1.14‒1.26		1.22	1.14‒1.31		1772	1.19	1.11‒1.27		1.20	1.09‒1.32
	Female breast								9443	0.86	0.84‒0.88		0.84	0.81‒0.88
Malignant neoplasm of genital organs														
	Uterus, not otherwise specified								5339	0.10	0.01‒0.78		0.51	0.05‒5.57
	Ovary, fallopian tube, and broad ligament								984	0.86	0.79‒0.94		0.88	0.78‒1.00
	Prostate gland	8005	1.01	0.98‒1.05		1.00	0.96‒1.05							
Malignant neoplasms of urinary tract														
	Bladder	3210	1.01	0.96‒1.06		1.04	0.97‒1.12		843	1.01	0.92‒1.11		1.01	0.89‒1.16
	Kidney	811	0.78	0.72‒0.86		0.73	0.64‒0.83		343	0.99	0.85‒1.15		1.18	0.95‒1.45
Malignant neoplasm of nervous system														
	Brain	568	1.09	0.97‒1.22		0.98	0.82‒1.18		286	1.06	0.90‒1.25		1.11	0.88‒1.40
	Thyroid gland	580	0.91	0.81‒1.01		0.80	0.68‒0.93		1689	1.03	0.96‒1.10		0.98	0.90‒1.08

^a^ The follow-up time period spanned from the initial insurance date (1 January 2000) until either the date of cancer diagnosis or the study end date (31 December 2018). ^b^ In Model 2, we excluded participants who had been newly diagnosed with solid tumors and had not been insured for >10 years, as well as participants who had been newly diagnosed with hematological tumors and had not been insured for ≥2 years. For solid tumors, the follow-up time period was defined as starting from the date of 10 years of insurance coverage and continuing until either the date of cancer diagnosis or the study end date (31 December 2018). For hematological tumors, the follow-up time period was defined as starting from the date of 2 years of insurance coverage and continuing until either the date of cancer diagnosis or the study end date (31 December 2018).

Table 3 presents the risks for various cancer types among Taiwanese farmers at different insurance periods. Regardless of whether the latency period is considered, early-insured farmers (2000–2004) had a higher risk of developing certain types of cancer, including overall cancer, lymphocytic leukemia, acute lymphocytic leukemia, CML, lymphoma, NHL, other lymphoid, multiple myeloma, oral cancer, lip cancer, esophagus cancer, rectum and rectosigmoid junction cancer, liver and intrahepatic bile duct cancer, lung cancer, and other non-melanoma skin cancer. However, only lymphoma, NHL, other lymphoid, and multiple myeloma, were found at different insurance periods.

**Table 3:** Hazard ratio (HR) and 95% confidence intervals (CI) for different cancer types among agricultural workers at different insurance period.

Sites/Types		2000–2004 Model 1 ^a^			2005–2009 Model 1 ^a^			2000–2004 Model 2 ^b^			2005–2009 Model 2 ^b^	
		HR	95% CI		HR	95% CI		HR	95% CI		HR	95% CI
Overall Cancer		1.05	1.04‒1.06		1.04	1.02‒1.07		1.06	1.05‒1.07		1.02	0.95‒1.09
Malignant neoplasm of lymphatic and haemopoietic tissue												
	Lymphocytic leukemia	1.10	1.03‒1.16		1.06	0.86‒1.31		1.11	1.04‒1.18		1.03	0.82‒1.28
	Acute myelogenous leukemia (AML)	1.13	1.03‒1.24		0.87	0.60‒1.26		1.12	1.01‒1.23		0.77	
	Acute lymphoblastic leukemia (ALL)	0.95	0.66‒1.36		1.98	0.18‒21.88		0.91	0.63‒1.32		1.98	0.18‒1.84
	Chronic myelogenous leukemia (CML)	1.19	1.00‒1.42		0.80	0.47‒1.35		1.21	1.01‒1.44		0.87	0.51‒1.51
	Chronic myelogenous leukemia (CLL)	0.97	0.80‒1.17		0.84	0.48‒1.48		1.05	0.86‒1.28		0.82	0.46‒1.48
	Other leukemia	1.08	0.98‒1.19		1.58	1.10‒2.26		1.10	0.99‒1.23		1.53	1.05‒2.23
	Lymphoma	1.05	0.99‒1.10		1.34	1.15‒1.58		1.07	1.01‒1.15		1.88	1.26‒2.81
	Hodgkin’s lymphoma	0.88	0.68‒1.14		1.32	0.58‒3.01		0.85	0.58‒1.26		1.10	0.07‒17.63
	Non-Hodgkin’s lymphoma (NHL)	1.05	1.00‒1.10		1.35	1.14‒1.58		1.08	1.01‒1.16		1.90	1.27‒2.85
	B-cell lymphomas	1.04	0.98‒1.11		1.28	1.04‒1.58		1.07	0.99‒1.16		1.68	1.02‒2.77
	Natural killer (NK)/T-cell lymphoma	0.95	0.82‒1.11		1.99	1.21‒3.30		0.95	0.76‒1.18		3.02	0.58‒15.73
	Other lymphoid	1.12	1.02‒1.23		1.28	0.93‒1.76		1.17	1.02‒1.34		2.26	1.05‒4.87
	Multiple myeloma	1.16	1.05‒1.29		1.36	0.96‒1.94		1.17	1.01‒1.36		3.43	1.35‒8.72
Malignant neoplasm of lip, oral cavity and pharynx												
	Oral	1.21	1.17‒1.25		1.08	0.96‒1.21		1.26	1.20‒1.32		1.09	0.81‒1.48
	Lip	1.37	1.19‒1.58		1.09	0.66‒1.80		1.53	1.26‒1.85		0.50	0.13‒1.95
	Larynx	1.00	0.92‒1.10		0.89	0.66‒1.20		1.09	0.96‒1.23		0.96	0.44‒2.08
	Major salivary glands	0.92	0.79‒1.09		1.70	0.99‒2.93		0.88	0.70‒1.10		1.16	0.29‒4.65
Malignant neoplasm of digestive organs and peritoneum												
	Esophagus	1.17	1.12‒1.23		0.95	0.80‒1.13		1.26	1.18‒1.35		1.12	0.71‒1.77
	Stomach	1.03	0.99‒1.07		1.01	0.88‒1.17		1.05	0.99‒1.11		0.84	0.58‒1.21
	Colon (excluding rectum)	0.96	0.93‒0.98		1.02	0.93‒1.12		0.96	0.93‒1.00		0.86	0.70‒1.07
	Rectum and rectosigmoid junction	1.12	1.08‒1.16		0.96	0.86‒1.07		1.10	1.05‒1.15		0.90	0.67‒1.21
	Sigmoid colon	0.98	0.94‒1.02		1.04	0.91‒1.19		0.97	0.92‒1.03		1.07	0.76‒1.51
	Anus, anal canal, and anorectum	0.62	0.47‒0.81		0.43	0.17‒1.13		0.70	0.48‒1.03		-	-
	Liver and intrahepatic bile duct	1.12	1.10‒1.15		1.09	1.01‒1.18		1.14	1.10‒1.18		1.10	0.89‒1.36
Malignant neoplasm of respiratory and intrathoracic organs												
	Lung	1.10	1.08‒1.12		1.00	0.92‒1.08		1.11	1.08‒1.15		1.03	0.85‒1.25
	Trachea and bronchi	1.15	0.98‒1.35		1.38	0.73‒2.64		1.19	0.90‒1.57		-	-
Malignant neoplasm of bone, connective tissue, skin and breast												
	Bones, joints, and articular cartilage	1.13	0.86‒1.48		0.86	0.36‒2.07		1.31	0.88‒1.97		1.23	0.17‒8.75
	Connective, subcutaneous and other soft tissues	0.96	0.85‒1.08		1.25	0.86‒1.82		0.93	0.79‒1.10		13.19	1.71‒101.51
	Melanoma of skin	1.41	1.14‒1.73		1.03	0.43‒2.48		1.34	0.98‒1.83		0.39	0.04‒3.77
	Other non-melanoma skin	1.19	1.14‒1.24		1.26	1.07‒1.50		1.21	1.14‒1.28		1.37	0.91‒2.05
	Female breast	0.85	0.83‒0.88		1.01	0.92‒1.10		0.85	0.82‒0.88		0.85	0.67‒1.08
Malignant neoplasm of genital organs												
	Uterus, not otherwise specified	0.11	0.01‒0.88		-	-		0.51	0.05‒5.57		-	-
	Ovary, fallopian tube, and broad ligament	0.86	0.79‒0.94		0.92	0.70‒1.21		0.90	0.79‒1.02		0.55	0.26‒1.17
	Prostate gland	1.00	0.97‒1.04		1.13	1.01‒1.26		1.00	0.95‒1.04		1.23	0.97‒1.55
Malignant neoplasms of urinary tract												
	Bladder	1.01	0.97‒1.06		0.95	0.80‒1.12		1.04	0.98‒1.11		0.84	0.57‒1.23
	Kidney	0.82	0.76‒0.90		0.94	0.73‒1.21		0.82	0.74‒0.92		1.29	0.68‒2.44
Malignant neoplasm of nervous system												
	Brain	1.09	0.99‒1.20		0.96	0.67‒1.38		1.04	0.90‒1.21		0.70	0.29‒1.66
	Thyroid gland	0.99	0.93‒1.05		1.04	0.86‒1.26		0.94	0.87‒1.02		0.81	0.48‒1.37

^a^ The follow-up time period spanned from the initial insurance date (January 1, 2000 or January 1, 2005) until either the date of cancer diagnosis or the study end date (December 31, 2018). ^b^ In Model 2, we excluded participants who had been newly diagnosed with solid tumors and had not been insured for >10 years, as well as participants who had been newly diagnosed with hematological tumors and had not been insured for ≥2 years. For solid tumors, the follow-up time period was defined as starting from the date of 10 years of insurance coverage and continuing until either the date of cancer diagnosis or the study end date (31 December 2018). For hematological tumors, the follow-up time period was defined as starting from the date of 2 years of insurance coverage and continuing until either the date of cancer diagnosis or the study end date (31 December 2018). ^d^ The data was analyzed only for female subjects. ^e^ The data was analyzed only for male subjects.

## Discussion

This study identified 136 913 incident cancer cases which can be used as a reference for regional health intervention policies for agricultural workers with cancer. Male farmers had a significantly higher risk for several types of cancer, including lymphocytic leukemia, CML, NHL, oral cancer, lip cancer, esophagus cancer, rectum and rectosigmoid junction cancer, liver and intrahepatic bile duct cancer, lung cancer, trachea and bronchi cancer and other non-melanoma skin cancer, even when considering the latency period. Female farmers had an elevated risk of multiple myeloma and other non-melanoma skin cancer. Moreover, this study found that lymphoma, NHL, other lymphoid, and multiple myeloma have an increased risk with the period of insurance enrollment.

This study revealed an increased risk of leukemia in male farmers, particularly in chronic CML and NHL, as well as an elevated risk of multiple myeloma in female farmers. Furthermore, the analysis found that lymphoma, NHL, other lymphoid, and multiple myeloma had a higher risk in recent insurance enrollment periods. Contact pesticides have been identified as a major factor contributing to the increased risk of NHL, multiple myeloma, and leukemia observed in agricultural populations (30). A meta-analysis of 13 case–control studies also reported that occupational pesticide exposure was associated with a significantly increased risk of NHL, as well as suggestive associations with other hematopoietic cancers (31). Similar findings have been reported in population-based case-control studies of pesticide exposure in Canada (32) and the United States (33). Exposure to specific insecticides, such as organophosphates crotoxyphos, dichlorvos, and famphur and the natural product pyrethrins and the chlorinated hydrocarbon methoxychlor, have been associated with a significantly increased risk of leukemia (odds ratio >2.0) (33). Although pesticide application exposure in farmland is a known fact, it is currently not possible to determine which specific active ingredients are involved in this study.

The present study observed that both male and female farmers had an elevated risk for non-melanoma skin cancer. Previous studies have suggested that intermittent intense and prolonged exposure to UVR is a significant risk factor for melanoma and non-melanoma skin cancer, particularly in the head and neck area (34–36). The increased risk in farmers is due to the prolonged and intense exposure to UVR during outdoor farming tasks (34). However, previous studies of female agricultural workers have not shown an increased risk for non-melanoma skin cancer (37–39). Although past studies of pesticide exposure among pesticide applicators have indicated associations between the type of pesticide use (maneb/mancozeb, parathion, carbaryl) and ever use of arsenical pesticides and cutaneous melanoma, non-melanoma skin cancer has not been mentioned (36).

In this study, it was found that male farmers in Taiwan had an increased risk of oral cancer, lip cancer, and esophagus cancer. Oral cancer is a multifactorial disease caused by several factors such as tobacco use, betel nut, alcohol consumption, and the presence of underlying pre-malignant diseases of the oral cavity, often in the context of a diet deficient in antioxidant vitamins and minerals (40). In Taiwan, the oral mucosa and tongue were the most common sites of oral cancer observed (41). Studies have shown that betel nut consumption without added tobacco can cause oral and esophageal cancers in humans in Asian regions (42, 43), and long-term consumption of betel nut among non-smokers has been associated with an increased risk of oral cancer (42). Betel nut is a highly profitable crop in Taiwan and is second most commonly cultivated crop after rice in the past. Additionally, many agricultural areas in Taiwan are close to aboriginal tribes consume a greater amount of betel nut. The increased risk of lip cancer is associated with outdoor occupations due to exposure to UVR, but smoking, betel nut chewing, and alcohol consumption cannot be ruled out as contributing factors (44).

This study found a significant protective effect of male farmers against colon cancer. Farmers typically have favorable risk factors for lower cancer risk in terms of physical activity and body weight (45). Previous research, such as the Agricultural Health Study (AHS), has reported that an increases in livestock populations is associated with a reduced risk of lung cancer (46). Moreover, endotoxins have been found to be negatively associated with lung cancer in various occupationally exposed populations, such as agricultural and textile workers (47). Mechanistic studies have shown that endotoxin can inhibit tumorigenesis and growth and stimulate the production of endogenous anti-tumor mediators (48, 49). In this study, male farmers with lung cancer had a higher risk during the early enrollment period, but not women. This could be attributed to higher smoking rates among early male farmers in Taiwan. However, there is currently no data on smoking rates among farmer populations in Taiwan.

This study used a cross-disciplinary approach and analyzed complementary medical claims data from the NHIRD covering all agricultural workers from FHI, which reduces the possibility of selection bias. This method is advantageous as it provides high statistical power and cost-free data collection, enabling systematic screening of diseases. A retrospective cohort study design was used to observe 136 913 incident cancer cases among 1 175 149 Taiwanese farmers, providing sufficient sample size of cancer cases to assess the effect of farmer exposure on cancer risk. Although the exposure assessment of this study is not as detailed as the AGRIculture and CANcer (AGRICAN), AHS, Canadian Census Health and Environment Cohorts (CanCHEC) studies, it offers valuable information to examine cancer patterns in specific populations, especially in Asian communities. This study provides a valuable addition to the cancer literature and should be investigated using more targeted studies.

The main limitation of this study is that farmers receive information on exposure to multiple occupational hazards, and the data are lacking on specific types of exposures experienced by farmers. A recent systematic evaluation study of pesticide exposure assessment methods used in occupational epidemiology studies found an increase in the use of self-reported exposure and work exposure matrices, and a decrease in job and registry assessments within the indirect methods category (50). Future work will include the establishment of an exposure matrix to evaluate the effects of specific exposures. Additionally, due to the unavailability of lifestyle data such as smoking, drinking, and betel nut chewing, we conducted the analysis by adjusting for area-level potential cancer predictors. The results showed that the risk of various cancers, including lymphocytic leukemia, other leukemia, lymphoma, non-Hodgkin’s lymphoma, multiple myeloma, oral, lip, esophagus, rectum and rectosigmoid junction, liver and intrahepatic bile duct, lung, and other non-melanoma skin cancer, was significantly higher among farmers compared to the general population (supplementare figure S1). We acknowledge the limitations of adjusting for risk factors at the area-level in the ecological regression analysis, as it introduces the possibility of ecological fallacy. This implies that the associations observed at the ecological level may not necessarily reflect the same associations at the individual level. However, even after accounting for risk factors at the area-level, our study consistently found associations between farmers and cancer outcomes. To provide more accurate and comprehensive findings, future studies should incorporate representative individual-level data samples. Unfortunately, our records of agricultural workers only date back to 2000. Before that, limited access to electronic data and documentation posed challenges in tracking cases of death, illness, or individuals who discontinued their insurance coverage. Consequently, there is a possibility that we may have missed cases prior to 2000, which could impact the completeness of our data and reduce the likelihood of capturing all cancer incidences among agricultural workers during that period.

### Concluding remarks

This study utilized a health insurance database analysis approach to identify patterns of cancer related to agriculture in Taiwan. The findings indicate that agricultural workers face an elevated risk of certain types of cancer, particularly hematological cancers. This heightened risk may be attributed to the unique occupational and environmental exposures associated with farm life and work. These results can inform future investigations into causal relationships and provide valuable insights into the specific agricultural exposures that may increase cancer risk.

### Ethics

The study was conducted according to the guidelines of the Declaration of Helsinki and approved by the Institutional Review Board of National Health Research Institutes (NIRB File Number: EC1061204-E).

## Supplementary material

Supplementary material
