# Supplementary material for: Cancer risks in a population-based study of agricultural workers: results from the Taiwan’s Farmers and Health Cohort study
Source: Scand J Work Environ Health. 2023 Aug 29;49(6):419–27. doi: 10.5271/sjweh.4106 (PMC10821750; doi:10.5271/sjweh.4106)
Supplement: Supplementary material [file SJWEH-49-419-S001.pdf]

Cancer risks in a population-based study of agricultural workers: results from the Taiwan's Farmers and Health Cohort study[1]

by Wei-Liang Chen, MD, PhD,1, 2 Gwan-Ling Lin, PhD,3 Yu-Jen Lin, MSC,4 Ting-Yao Su, PhD,4 Chung-Ching Wang, MD, PhD,1, 2 Wei-Te Wu, PhD

1. *Supplementary material*
2. *Correspondence to: Wei-Te Wu, PhD, National Institute of Environmental Health Sciences National Health Research Institutes, 35 Keyan Road, Zhunan, Miaoli County, 35053 Taiwan, ROC. [E-mail: ader.una@gmail.com]*

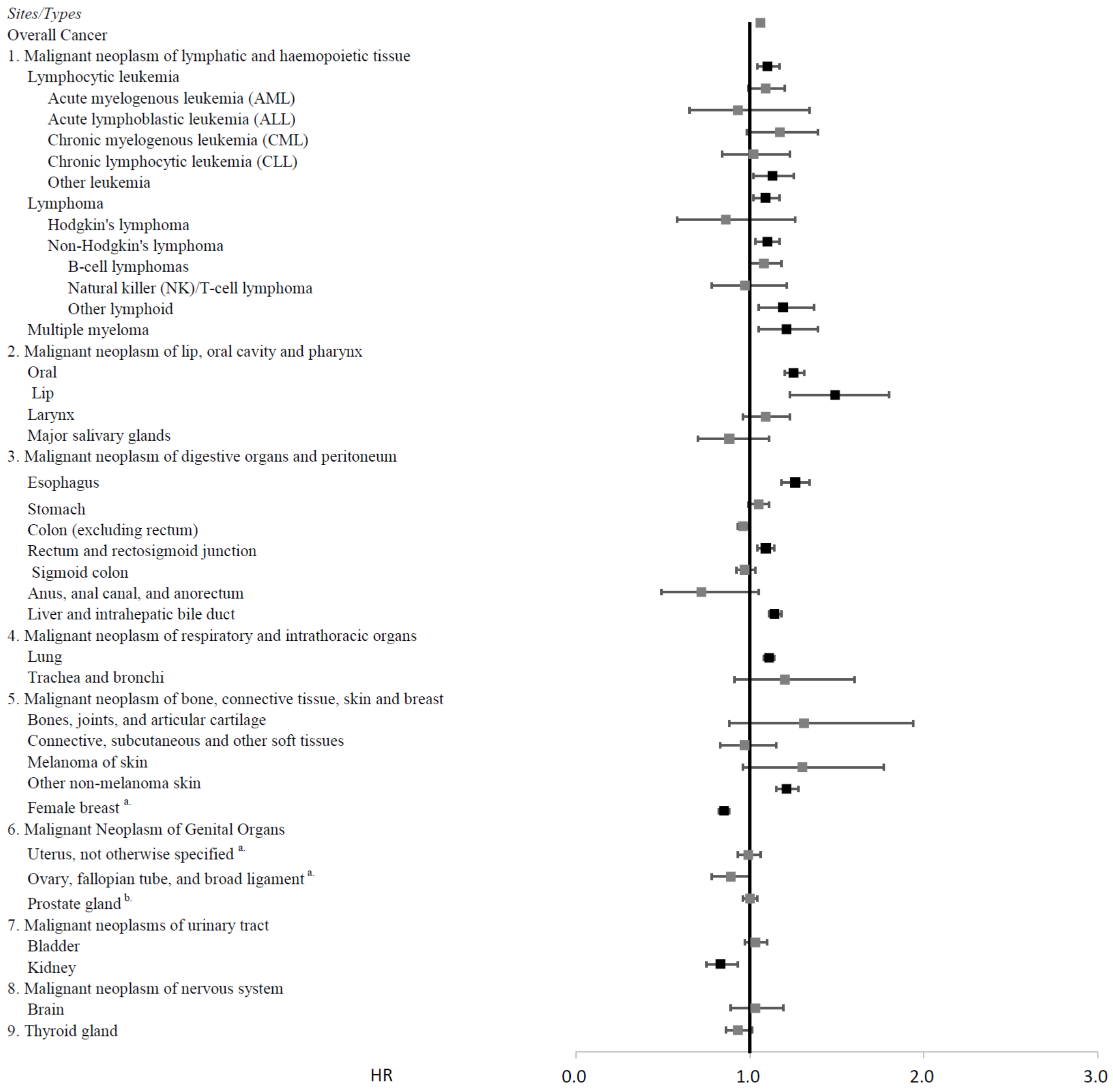

Figure S1 Adjusted Hazard Ratio and 95% confidence intervals (CI) for cancer types in agricultural workers. * Adjusted for smoking rate, drinking rate, betel nut chewing rate, number of patients served by physicians, number of chronic beds per 10,000 populations, mammography screening rate (only in breast cancer analysis), oral mucosa examination utilization rate (only in oral and oropharyngeal cancer analysis), and ultraviolet ray exceedance rate (only in non-melanoma cancer analysis) for counties and cities

Table S1 Target outcomes based on the ICD-O-3 and the 2008 WHO classification of lymphoid neoplasms

Sites/Types	2008 WHO Classification / ICD-O-3 Codes
Overall Cancer	C00–C80
1. Malignant neoplasm of lymphatic and haemopoietic tissue	
Lymphocytic leukemia	C91 (M-98003 – M-99643, M-99803 – M-99893)
Lymphoma	C77 (M8000 – M9589)
Hodgkin's lymphoma	C77 (M96503/96513/96523/96533/96593/96613/96623/96633/96643/96653/96673)
Non-Hodgkin's lymphoma	C77 (M-95903 – M-95963, M-96703 – M-97293, M-97503 – M-97583, M-97643)
Multiple myeloma	C90.0 (M-9731–M-9732, M9734)
2. Malignant neoplasm of lip, oral cavity and pharynx	
Oral	C00–C06
Lip	C00
Larynx	C32
Major salivary glands	C07–C08
3. Malignant neoplasm of digestive organs and peritoneum	
Esophagus	C15
Stomach	C16
Colon (excluding rectum)	C18
Rectum and rectosigmoid junction	C19–C20
Sigmoid colon	C187
Anus, anal canal, and anorectum	C21
Liver and intrahepatic bile duct	C22

4. Malignant neoplasm of respiratory and intrathoracic organs	
Lung	C34
Trachea and bronchi	C339, C340
5. Malignant neoplasm of bone, connective tissue, skin and breast	
Bones, joints, and articular cartilage	C40–C41
Connective, subcutaneous and other soft tissues	C47, C49
Melanoma of skin	C44 (M-87203 – M-87903)
Other non-melanoma skin cancer	C44 (excluding M-87203 – M-87903)
Female breast	C50
6. Malignant Neoplasm of Genital Organs	
Uterus, not otherwise specified	C55
Ovary, fallopian tube, and broad ligament	C56, C570–C574
Prostate gland	C61
7. Malignant neoplasms of urinary tract	
Bladder	C67
Kidney	C64
8. Malignant neoplasm of nervous system	
Brain	C71
9. Thyroid gland	C73

* ICD-O-3, International Classification of Diseases for Oncology.

Table S2. Information on the overall description of the area-level variables

Insurance Area	Smoking rate (%)	Drinking rate (%)	Betel nut chewing rate (%)	Mammography screening rate (45-69 years old) (%)	Oral mucosa examination utilization rate (30-69 years old) (%)	Number of patients served by physicians	Number of chronic beds per 10 000 populations	Ultraviolet ray exceedance rate (%)
Penghu County	16.1	27.1	3.4	37.4	71.2	767.9	7.6	74.0
Kinmen County	15.1	-	1.8	18.5	14.7	1,582.7	4.3	73.2
Keelung City	16.5	50.4	2.3	45.6	33.6	490.3	10.1	55.9
Taipei City	8.1	55.8	1.8	41.9	53.5	257.9	2.3	61.6
Hsinchu City	10.9	44.1	1.7	45.9	35.0	507.6	1.1	66.6
Hsinchu County	18.6	42.4	4.2	43.3	43.0	991.1	9.9	66.6
Miaoli County	17.8	38.0	5.3	32.4	35.6	936.6	10.1	77.0
Yilan County	18.5	35.9	3.5	45.7	51.8	667.5	15.2	65.8
Taoyuan City	10.6	44.7	2.8	42.6	47.9	551.2	6.4	68.8
Taitung County	17.6	52.9	11.0	38.7	51.7	734.6	7.7	75.3
Nantou County	22.5	32.4	6.1	36.8	43.0	770.6	17.7	71.8
Changhua County	13.4	35.5	3.6	40.5	66.1	619.1	9.7	80.8
New Taipei City	13.6	47.4	2.7	45.8	49.5	714.4	4.7	58.6
Chiayi County	15.0	33.7	6.3	41.0	52.6	639.4	9.1	82.7
Chiayi City	14.4	37.1	2.3	42.9	59.2	296.8	18.2	82.2
Kaohsiung City	12.0	36.7	3.0	39.1	52.5	430.6	5.3	78.4
Yunlin County	12.9	37.6	3.4	29.5	41.2	775.2	4.7	80.3
Taichung City	11.2	48.0	2.6	37.1	59.3	448.4	7.9	68.5
Pingtung County	16.9	30.5	5.6	35.6	60.2	712.2	8.5	85.8
Tainan City	11.8	40.7	1.9	40.3	57.6	511.2	5.2	84.7
Hualien County	15.9	47.3	8.1	36.1	80.3	384.5	51.2	66.8

Table S3 The numbers of cancer cases and corresponding hazard ratio and 95% CI for cancer types among agricultural workers

	Agricultural Workers	General Population Controls	Model 1 (Ref.: Controls)		Model 2 (Ref.: Controls) [c.]	
Sites/Types	*cases*	*cases*	*HR*	*95%CI*	*HR*	*95%CI*
Overall Cancer	136 913	130 248	1.05	1.04–1.06	1.06	1.05–1.07
1. Malignant neoplasm of lymphatic and haemopoietic tissue						
Lymphocytic leukemia	2384	2179	1.09	1.03–1.16	1.10	1.04–1.17
Acute myelogenous leukemia (AML)	938	842	1.11	1.01–1.22	1.09	0.99–1.20
Acute lymphoblastic leukemia (ALL)	59	61	0.96	0.67–1.38	0.93	0.65–1.34
Chronic myelogenous leukemia (CML)	302	264	1.14	0.97–1.35	1.17	0.98–1.39
Chronic myelogenous leukemia (CLL)	232	243	0.95	0.80–1.14	1.02	0.84–1.23
Other leukemia	853	769	1.11	1.01–1.22	1.13	1.02–1.25
Lymphoma	3918	3669	1.07	1.02–1.12	1.09	1.02–1.17
Hodgkin’s lymphoma	118	129	0.91	0.71–1.17	0.86	0.58–1.26
Non-Hodgkin’s lymphoma (NHL)	3800	3540	1.07	1.03–1.12	1.10	1.03–1.17
B-cell lymphomas	2344	2216	1.06	0.99–1.12	1.08	1.00–1.18
Natural killer (NK)/T-cell lymphoma	377	371	1.02	0.88–1.17	0.97	0.78–1.21
Other lymphoid	1,079	953	1.13	1.04–1.24	1.19	1.05–1.37
Multiple myeloma	838	711	1.18	1.07–1.30	1.21	1.05–1.39
2. Malignant neoplasm of lip, oral cavity and pharynx						
Oral	8194	6809	1.20	1.16–1.24	1.25	1.20–1.31
Lip	481	357	1.35	1.17–1.54	1.49	1.23–1.80
Larynx	1090	1095	1.00	0.92–1.08	1.09	0.96–1.23
Major salivary glands	314	322	0.97	0.83–1.14	0.88	0.70–1.11
3. Malignant neoplasm of digestive organs and peritoneum						
Esophagus	3790	3275	1.16	1.10–1.21	1.26	1.18–1.34

Stomach	6115	5945	1.03	0.99–1.07	1.05	0.99–1.11
Colon (excluding rectum)	11 549	12 046	0.96	0.94–0.98	0.96	0.93–0.99
Rectum and rectosigmoid junction	8197	7408	1.11	1.07–1.14	1.09	1.04–1.14
Sigmoid colon	4984	5059	0.99	0.95–1.03	0.97	0.92–1.03
Anus, anal canal, and anorectum	92	153	0.60	0.46–0.78	0.72	0.49–1.05
Liver and intrahepatic bile duct	19 666	17 523	1.12	1.10–1.14	1.14	1.11–1.18
4. Malignant neoplasm of respiratory and intrathoracic organs						
Lung	17 792	16 293	1.09	1.07–1.12	1.11	1.08–1.14
Trachea and bronchi	348	297	1.17	1.00–1.36	1.20	0.91–1.60
5. Malignant neoplasm of bone, connective tissue, skin and breast						
Bones, joints, and articular cartilage	121	110	1.10	0.85–1.42	1.31	0.88–1.94
Connective, subcutaneous and other soft tissues	591	602	0.98	0.88–1.10	0.97	0.83–1.15
Melanoma of skin	223	161	1.38	1.13–1.69	1.30	0.96–1.77
Other non-melanoma skin cancer	5041	4231	1.19	1.14–1.24	1.21	1.15–1.28
Female breast [a.]	9443	10 946	0.86	0.84–0.89	0.85	0.82–0.88
6. Malignant Neoplasm of Genital Organs						
Uterus, not otherwise specified [a.]	5339	5386	1.03	0.99–1.07	0.99	0.93–1.06
Ovary, fallopian tube, and broad ligament [a.]	984	1135	0.87	0.80–0.94	0.89	0.78–1.00
Prostate gland [b.]	8005	7931	1.01	0.98–1.04	1.00	0.96–1.04
7. Malignant neoplasms of urinary tract						
Bladder	4053	4016	1.01	0.97–1.05	1.03	0.97–1.10
Kidney	1154	1383	0.84	0.77–0.90	0.83	0.75–0.93
8. Malignant neoplasm of nervous system						
Brain	854	791	1.08	0.98–1.19	1.03	0.89–1.19
9. Thyroid gland	2269	2283	0.99	0.94–1.05	0.93	0.86–1.01

[a.] The data was analyzed only for female subjects. [b.] The data was analyzed only for male subjects.

[c.] In Model2, we excluded participants who had been newly diagnosed with solid tumors and had not been insured for more than 10 years, as well as participants who had been newly diagnosed with hematological tumors and had not been insured for at least 2 years.
